# Characterisation of phosphorylated nucleotides by collisional and electron‐based tandem mass spectrometry

**DOI:** 10.1002/rcm.7701

**Published:** 2016-09-05

**Authors:** Andrew T. Ball, Aruna S. Prakash, Anthony W.T. Bristow, Martin Sims, Jackie A. Mosely

**Affiliations:** ^1^ Department of ChemistryDurham UniversitySouth RoadDurhamDH1 3LEUK; ^2^ Global Medicines DevelopmentAstraZenecaMacclesfieldSK10 2NAUK; ^3^ Pharmaceutical Sciences, Innovative Medicines and Early DevelopmentAstraZenecaMacclesfieldSK10 2NAUK

## Abstract

**Rationale:**

Tandem mass spectrometry of phosphorylated ions can often yield a limited number of product ions owing to the labile nature of phosphate groups. Developing techniques to improve dissociation for this type of ion has implications for the structural characterisation of many different phosphorylated ions, such as those from nucleotides, pharmaceutical compounds, peptides and polymers.

**Methods:**

Solutions of adenosine monophosphate, diphosphate and triphosphate (AMP, ADP and ATP) were studied in a hybrid linear ion trap–Fourier transform ion cyclotron resonance (FTICR) mass spectrometer. Precursor ions with an overall single positive charge, including protonated nucleotides or nucleotide cations containing one, two or three sodium atoms, were isolated for tandem mass spectrometry. Collision‐induced dissociation (CID) was performed in the linear ion trap, with electron‐induced dissociation (EID) being conducted in the FTICR cell.

**Results:**

EID resulted in many product ions not seen in CID. EID product ion spectra were seen to vary for AMP, ADP and ATP when the nucleotide cation contained zero, one, two or three sodiums. Precursor cations that contain two or three sodiums mainly formed product ions derived from the phosphate group. Conversely, when a precursor ion containing no sodium underwent EID, product ions mainly relating to the non‐phosphate end of the ion were observed. The number of phosphate groups was not seen to greatly affect either CID or EID product ion spectra.

**Conclusions:**

The presence of sodium in a precursor ion directs electron‐induced bond dissociation, thus enabling targeted, and therefore tuneable, fragmentation of groups within that precursor ion. For all precursor ions, the most useful product ion spectra were obtained by EID for a precursor ion containing one sodium, with bond dissociation occurring across the entire nucleotide cation. The findings of this study can be used to improve the structural elucidation of many phosphorylated molecules by broadening the range of product ions achievable. © 2016 The Authors. *Rapid Communications in Mass Spectrometry* Published by John Wiley & Sons Ltd.

Electron‐capture dissociation (ECD) is an electron‐based dissociation technique that has been widely adopted for the structural characterisation of biological macromolecules. It uses low‐energy electrons (typically 1–5 eV), which interact with multiply charged analyte ions in Fourier transform ion cyclotron resonance mass spectrometers.^1^, ^2^, ^3^, ^4^ In essence, an electron is captured by the analyte ion, forming an excited charge‐reduced radical cation that then dissociates.^2^, ^5^, ^6^ Unimolecular dissociation by this method is able to provide complementary fragmentation information to collision‐induced dissociation (CID) and infrared multiphoton dissociation (IRMPD), retaining post‐translational biological modifications that are otherwise lost and providing protein sequence information from intact proteins.^1^, ^2^, ^7^, ^8^, ^9^ The analysis of synthetic polymers also benefits from ECD as fewer complex rearrangements allow for easier characterisation.^10^, ^11^ Hot electron‐capture dissociation (hECD) was developed by Kjeldsen *et al.* where higher energy electrons (10 eV) were used for the analysis of multiply charged polypeptides, resulting in increased fragmentation compared with ECD.^12^, ^13^ More recently, electron‐induced dissociation (EID) was developed, which allows for the study of singly charged positive and negative ions. Here, electrons with a high kinetic energy (10–30 eV) interact with precursor ions causing concurrent ionisation and excitation.^14^, ^15^, ^16^ EID has been shown to yield product ions as the result of vibrational and electronic dissociation for a wide range of singly charged ions, including peptides, where cleavage is induced along the backbone and at side‐chain groups, forming product ions that are observed in CID and ECD.^15^, ^16^, ^17^, ^18^ EID has proved useful for the structural elucidation of protonated pharmaceutical compounds, as well as sodium, potassium and ammonium adducts, with EID providing complementary information to CID, thus allowing for better characterisation of these ions.^19^ Similarities can be drawn between the product ions formed by EID of the protonated and ammonium‐adducted precursors, although a small amount of extra structural information could be observed for the ammonium adduct. The alkali‐metal‐adducted precursors formed more product ions than the protonated and ammonium‐adducted precursors, with sodium‐containing precursor ions providing the most structural information.^20^ Metal‐adducted polyketide precursor ions have also been studied by EID and CID, with lithium‐adducted precursor ions resulting in the most structural information. Although product ions were similar for all techniques, the most abundant were formed by EID.^21^ EID, CID and IRMPD have also been compared for the study of chlorophyll‐*a* where EID was shown to provide complementary product ions to CID. It was noted that loss of H^•^ was common from EID along with fragmentation resulting in odd‐election species.^22^ DNA and RNA have been studied by electron‐detachment dissociation (EDD), an electron‐based fragmentation technique used for multiply charged anions. When compared with CID and IRMPD, EDD resulted in minimal base loss and no secondary fragmentation. Determination of the binding position for Os complexes to DNA was also possible due to Os‐bound product ions being formed by EDD.^23^, ^24^, ^25^, ^26^, ^27^, ^28^ Flosadóttir *et al.* 29 observed the effect of exchanging up to seven protons with sodium ions on bond cleavages by matrix‐assisted laser desorption/ionisation (MALDI) for deoxy‐oligonucleotides. Here, backbone cleavages were greatly reduced with increasing sodium content. Conversely, the neutral loss of certain bases increased with a greater number of sodium atoms on the precursor, thought to be owing to protonation of an adjacent base with higher proton affinity. Unique product ions were formed with the inclusion of sodium atoms, such as a complex fragmentation pathway involving the loss of several central bases and recombination to form a product ion.^29^

Phosphorylated ions have predictable vibrational tandem mass spectrometric behaviour, with loss of phosphate groups predominantly observed owing to the labile nature of the bond.^30^, ^31^, ^32^, ^33^, ^34^ Cyclic adenosine monophosphate (cAMP) analogues have been studied by CID, with limited product ions formed. The majority of product ions related to cleavage between the ribose‐phosphate and aminopurine sub‐structures.^30^ The limited nature of the product ions formed from CID for this type of ion has led to studies aimed at gaining increased information from tandem mass spectrometry, including the use of electron‐based techniques. ECD of adenosine triphosphate (ATP) and analogous ions was shown to yield complementary product ions to CID for doubly charged precursor ions. Unique to ECD, ribose cross‐ring cleavage was observed as well as several hydrated product ions formed by cross‐ring cleavages at other groups. As CID also yields unique product ions, Liu *et al.* concluded that a combination of ECD and CID can be implemented to gain maximum product ion information for acidic metabolites.^35^ ECD also results in high sequence coverage for phosphopeptides containing up to four phosphorylation sites.^31^ The effect of increasing the number phosphate groups has on sequence coverage by ECD was that for [M + 2H]^2+^, increased levels of phosphorylation results in lower sequence coverage of the peptides. This is thought to be caused by the phosphate groups forming salt bridges with amino acid side chains.^7^ Losses usually observed by CID, such as H_2_O, phosphate groups and phosphoric acid, were not observed by ECD.^21^

In this study, adenosine monophosphate diphosphate and triphosphate (AMP, ADP and ATP) have been analysed using CID and EID with varying numbers of sodium atoms contained within the precursor ion. The aim was to observe the effect of increasing numbers of phosphate groups and increasing number of sodium cations upon the two types of dissociation, and then use that information to gain maximum structural information and to determine the extent to which desired or directed fragmentation could be achieved through design.

## Experimental

### Sample preparation

AMP, ADP and ATP were purchased from Sigma‐Aldrich Company Ltd (Gillingham, UK). All samples were made up to 1 μg mL^−1^ in 50:50 HPLC‐grade acetonitrile from Fisher Scientific (Loughborough, UK) and deionised water (18.2 MΩ cm) with 0.1% formic acid from Sigma‐Aldrich.

### Mass spectrometry

All mass spectrometric measurements were performed on a hybrid linear ion trap–Fourier transform‐ion cyclotron mass spectrometer equipped with a 7.0 T superconducting magnet (LTQFT from ThermoFinnigan, Bremen, Germany). Sample solutions were infused directly into an electrospray ionisation (ESI) source at 5 μL min^−1^, from which positive ions were chosen for analysis. A heated capillary was set to 350°C and a sheath gas (nitrogen) optimised to a flow rate that delivered a stable spray. The spray voltage was 4 kV and the tube lens voltage was optimised to give the most intense precursor ion signal. For tandem mass spectrometry the isolation window was set 4 u. CID was performed in the ion trap region of this hybrid instrument using helium as the collision gas with 20–30 eV normalised collision energy, with the product ions measured in the FTICR instrument. For EID, the indirectly heated dispenser cathode inside the FTICR cell generated electrons at 26.5 eV for 70 ms. All data were recorded and processed using Xcalibur software (version 2.0; ThermoFinnigan, San Jose, CA, USA). Data were internally calibrated from the precursor ion and the common product ion [H_4_PO_4_]^+^.

## Results and Discussion

### Terminology

The overall charge state for each nucleotide cation studied was 1+, yet such ions with a net single positive charge could be thought of in a number of ways. This paper has employed the terminology of protonated nucleotide to refer to the cation of a nucleotide that only contains the atoms C, H N O and P and must be protonated (e.g. [C_10_H_14_N_5_O_7_P + H^+^] for AMP). The terminology ‘monosodium nucleotide’ refers to the cation, which could be either a sodiated nucleotide with OH groups, or a protonated nucleotide that has an ONa group, e.g. [C_10_H_14_N_5_O_7_P + Na^+^] or [C_10_H_13_N_5_O_7_PNa + H^+^], respectively, for AMP. The terminology ‘disodium nucleotide’ is used to encompass both the possibility of a sodiated nucleotide with one ONa group and OH groups, and a protonated nucleotide that has two ONa groups; the terminology ‘trisodium nucleotide’ encompasses both sodiated nucleotide with two ONa groups and a protonated nucleotide that has three ONa groups.

### EID and CID of protonated AMP, monosodium AMP and disodium AMP

**Figure 1:**
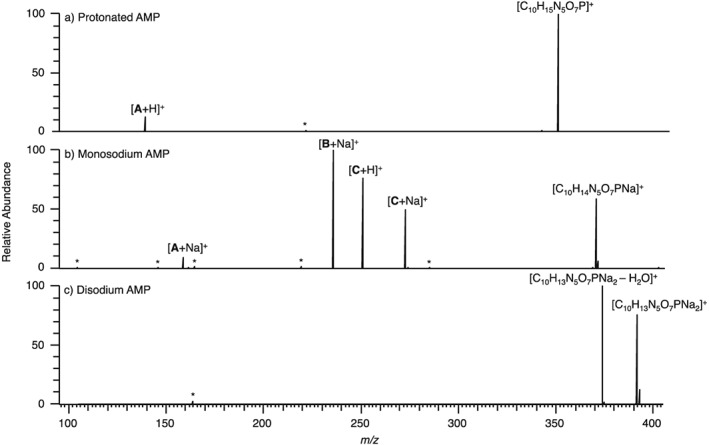
CID of precursor ions with an overall single charge for (a) protonated AMP, (b) monosodium AMP, and (c) disodium AMP. A full list of *m/z* values and corresponding molecular formulae are given in the Supporting Information. * indicates instrument noise.

**Figure 2:**
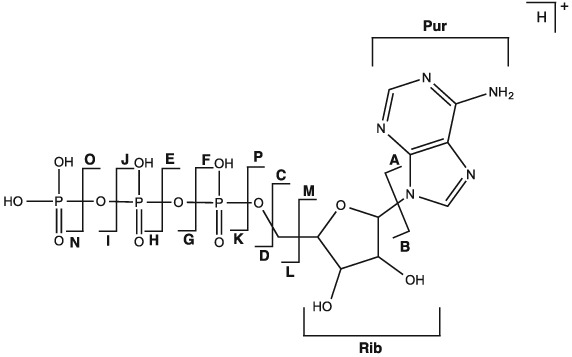
Diagram of protonated ATP describing the nomenclature used to identify a particular bond cleavage and corresponding product ion, plus the terminology used to discuss cleavages involving the purine (**Pur**) and ribose (**Rib**) regions. By extension, this diagram also applies to AMP and ADP and the mono‐, di‐ and trisodium species.

CID of protonated AMP (Fig. 1(a)) yielded only one product ion at *m/z* 136.0617, relating to cleavage **A**, [C_5_H_6_N_5_]^+^, with an error of −0.2 ppm (Fig. 2). When monosodium AMP underwent CID (Fig. 1(b)), cleavage at **A** occurred again, with a sodium‐containing ion being produced: [C_5_H_5_N_5_Na]^+^ at *m/z* 158.0436 (−0.5 ppm). Cleavage **B** resulted in an ion at *m/z* 234.9978, again with the inclusion of sodium, [C_5_H_9_O_7_PNa]^+^, with an error of 0.0 ppm. This indicates that the sodium cation can be retained by either fragment of the monosodium AMP precursor ion although the relative peak intensities would suggest that the phospho‐ribose region has the stronger affinity for the sodium cation. Two other product ions, formed by cleavage **C**, were also observed (*m/z* 250.0934 and 272.0753) one with and one without the sodium atom, [C_10_H_12_N_5_O_3_]^+^ and [C_10_H_11_N_5_O_3_Na]^+^, with errors of −0.4 and −0.3 ppm, respectively. The relative abundance of [C_10_H_12_N_5_O_3_]^+^ to [C_10_H_11_N_5_O_3_Na]^+^ would suggest that the more favourable location for the sodium is on the phosphate. Conversely, disodium AMP is a very stable cation, only able to lose water by CID, as shown in Fig. 1(c) (*m/z* 374.0235, [C_10_H_11_N_5_O_6_PNa_2_]^+^, −0.3 ppm).

**Figure 3:**
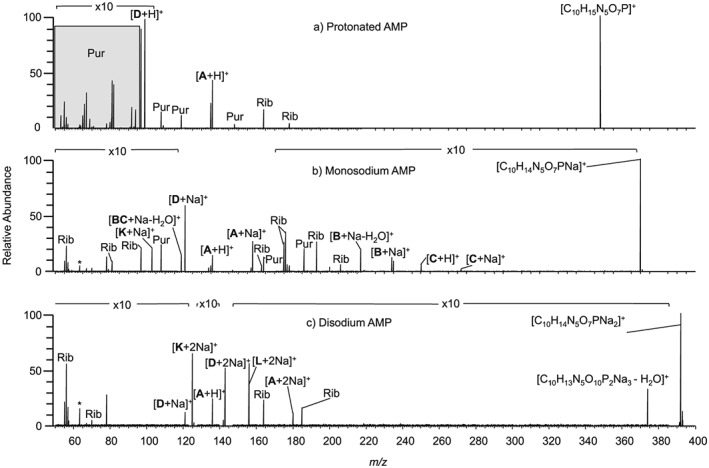
EID of precursor ions with an overall single charge for (a) protonated AMP, (b) monosodium AMP, and (c) disodium AMP. A full list of *m/z* values and corresponding molecular formulae is given in the Supporting Information. * indicates instrument noise.

In contrast to CID, EID of protonated AMP resulted in the formation of significantly more product ions, as shown in Fig. 3. When protonated AMP (Fig. 3(a)) undergoes EID, over 80% of the product ions relate to cleavage at the aminopurine group. Cross‐ring cleavages are only observed by EID for this precursor ion, such as cleavages across the aminopurine (**Pur**) and ribose (**Rib**) groups. There is precedence for this as Liu *et al.* noted that cross‐ring cleavage for the ribose group of metabolites including ATP was only observed by ECD.^35^ For protonated AMP, either the phosphate group or the aminopurine group can retain the proton, as demonstrated by cleavages **A** (*m/z* 136.0618, [C_5_H_6_N_5_]^+^, 0.0 ppm) and **D** (*m/z* 98.9841, [H_4_PO_4_]^+^, −0.4 ppm).

EID of monosodium AMP (Fig. 3(b)) resulted in significantly more product ions than seen by CID, with over 75% pertaining to cleavage at the ribose group. The cross‐ring cleavages at the aminopurine group seen for EID of protonated AMP are less numerous for EID of monosodium AMP, with an increased number across the ribose group (**Rib**) being observed. Some cleavages are common to EID of protonated AMP, such as **A** and **D**. **A** is seen with and without sodium (*m/z* 136.0618 and 158.0438, [C_5_H_6_N_5_]^+^ and [C_5_H_5_NNa_5_]^+^, 0.1 and 0.4 ppm) whereas **D** is seen exclusively containing sodium (*m/z* 120.9661, [H_3_PO_4_Na]^+^, 0.1 ppm), suggesting that the phosphate group has a higher affinity for sodium cations. Cleavage **bc** relating to the central methyl‐ribose group was observed with and without sodium (*m/z* 119.0104, [C_5_H_4_O_2_Na]^+^, 0.1 ppm and *m/z* 97.0284, [C_5_H_5_O_2_]^+^, −0.1 ppm), showing that the ribose group is also capable of retaining a sodium cation. **BC** was not formed by EID of protonated AMP, which demonstrates that the location of the sodium causes this bond cleavage.

For disodium AMP (Fig. 3(c)), EID yielded fewer product ions than seen for protonated AMP and monosodium AMP. Increased cleavage at the phosphate group was observed, with over 50% of all the product ions relating to this region of the precursor ion. Cross‐ring cleavages were only seen to occur at the ribose group (**Rib**). Two sodium cations are seen to reside on the phosphate group as well as independently on the aminopurine group following cleavages **D** and **A**, respectively. **A** is seen with zero and two sodiums (*m/z* 136.0618, [C_5_H_6_N_5_]^+^, 0.1 ppm and *m/z* 180.0257, [C_5_H_4_N_5_Na_2_]^+^, 0.3 ppm) whereas **D** is seen with one and two sodiums (*m/z* 120.9661, [H_3_PO_4_Na]^+^, −0.1 ppm and *m/z* 142.9481, [H_2_PO_4_Na_2_]^+^, 0.0 ppm) and **L** only with two sodiums (*m/z* 155.9559, [CH_3_PO_4_Na_2_]^+^, 0.1 ppm), lending further evidence that the aminopurine group has a stronger proton affinity than the phosphate group. Non‐covalent cation‐π interactions have been reported for tryptophan and other aromatic molecules and indicate that cations such as sodium can be tightly bound to the aromatic regions of a molecule in the gas phase, supporting this assignment.^36^, ^37^ The precursor ion was observed to lose water (*m/z* 374.0236, [C_10_H_11_N_5_O_6_PNa_2_]^+^, 0.1 ppm) as previously seen by CID, but not by ECD, supporting the conclusion that vibrational dissociation is a factor in EID.^18^ In each case EID was able to generate a greater degree of information than CID, but as with CID the richest spectra were obtained from the monosodium species. Table 1 summarises the bonds cleaved in AMP, AMP monosodium and AMP disodium. As more sodium is included with the precursor ion, dissociation is clearly biased towards the phosphate group.

**Table 1:** Summary of product ions formed by CID and EID for all precursor ions

Compound	Adduct	Cleavages from EID	Cleavages from CID
**AMP**	H	Purine(15), Ribose(2), **A** , **D**	**A**
	**Na**	Purine(4), Ribose(8), **M** , **A** , **A** *, **B** *, [ **B** *‐H_2_ O], [ **BC** *‐H_2_ O], **D** *, **K** *, **M** *	**A** , **C** , **B** *, **C** *
	**2Na**	Ribose(4), **A** *, **D** *, **A** **, **D** **, **K** **, **L** **	‐H_2_ O
**ADP**	H	Purine(8), Ribose(4), **A** , **C** , **D** , **E**	[ **C** ‐H_2_ O], [ **E** ‐H_2_ O], **E**
	**Na**	Purine(2), Ribose(14), **A** , [ **BC** ‐H_2_ O], **M** , **A** *, **B** *, [ **B** *‐H_2_ O], **BE** *, **BF** *, **D** *, **K** *, **L** *	**C** , **F** , **B** *, **BF** *, [ **B** *‐H_2_ O], **D** *
	**2Na**	Ribose(7), **A** , **M** , **A** *, **B** *, [ **B** *‐H_2_ O], **BE** *, **BF** *, **D** *, **G** *, **H** *, **K** *, **L** *, **G** **	**B** **, [ **B** **‐H_2_O], **BF** **, **D** **, **F** **
	**3Na**	Ribose(3), **A** , **G** , **F** **, **G** **, **H** **, **D** ***, **K** ***	‐H_2_ O, **F** **
**ATP**	H	Purine(5), Ribose(4), **A** , **BC** , [ **BC** ‐H_2_ O], **G** , **H** , **I**	**E** , **K** , [ **K** ‐H_2_ O]
	**Na**	Purine(3), Ribose(7), **A** , **BC** , **C** , **G** , **I** , **B** *, [ **B** *‐H_2_ O], **BF** *, **BK** *, **D** *, **G** *, **I** *, **JB** *, **K** *, **L** *, **N** *	**B** *, [ **B** *‐H_2_ O], **C** *, **J** *
	**2Na**	Purine(1), Ribose(11), **A** , **C** , **A** *, **I** *, **G** *, **B** **, [ **B** **‐H_2_ O], **D** **, **F** **, **G** **, **H** **, **I** **, **J** **, **JB** **, **K** **, **L** **, **N** **	**B** **, [ **B** **‐H_2_ O], **D** **, **J** **
	**3Na**	Purine(2), Ribose(8), **A** , **A** *, **A** **, **F** **, **G** **, **H** **, **I** **, **N** **, **B** ***, [ **B** ***‐H_2_ O], **D** ***, **G** ***, **H** ***, **I** ***, **J** ***, **JB** ***, **K** ***, **L** ***	**B** ***, [ **B** ***‐H_2_ O], **J** ***, **JB** ***

* Denotes the sodium content for each product ion (* = 1; ** = 2 and *** = 3).

### EID and CID of protonated ADP, monosodium ADP, disodium ADP and trisodium ADP

**Figure 4:**
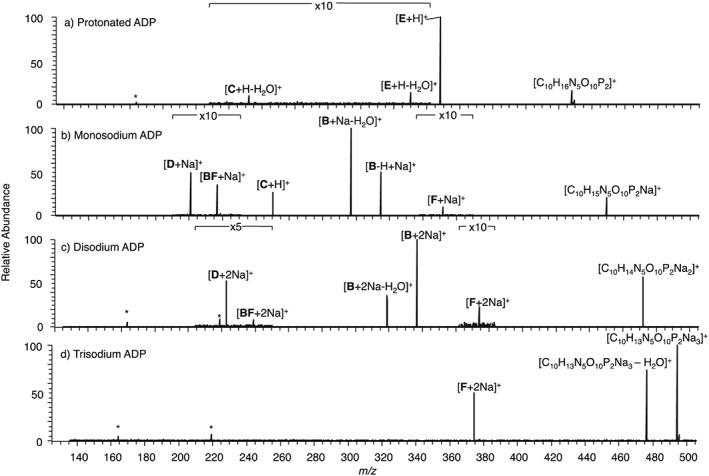
CID of precursor ions with an overall single charge for (a) protonated ADP, (b) monosodium ADP, (c) disodium ADP, and (d) trisodium ADP. A full list of *m/z* values and corresponding molecular formulae is given in the Supporting Information. * indicates instrument noise.

**Figure 5:**
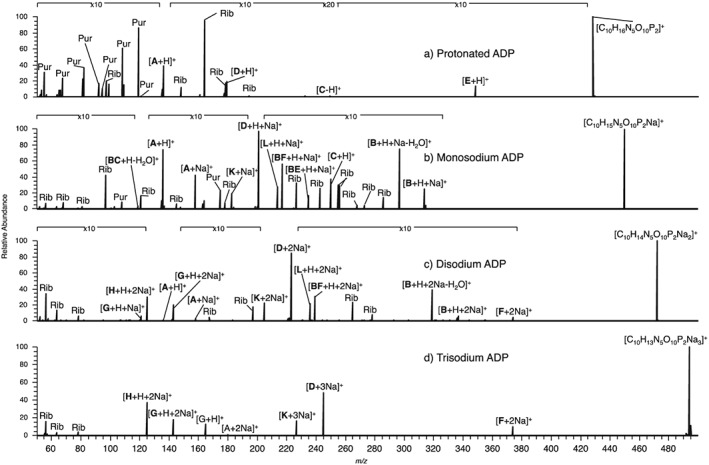
EID of precursor ions with an overall single charge for (a) protonated ADP, (b) monosodium ADP, (c) disodium ADP, and (d) trisodium ADP. A full list of *m/z* values and corresponding molecular formulae is given in the Supporting Information. * indicates instrument noise.

Figure 4 shows the CID product ion spectra for protonated, monosodium, disodium and trisodium ADP with the monosodium species providing the most information, analogous to AMP. CID of protonated ADP (Fig. 4(a)) yielded only product ions related to dissociation at one or both phosphate groups. As with protonated AMP, EID of protonated ADP (Fig. 5(a)) yielded many more product ions than CID, with most relating to extensive cleavage at the aminopurine group. Cross‐ring cleavages at the aminopurine (**Pur**) and ribose (**Rib**) groups are common, as well as some cleavage along the phosphate backbone. As the majority of product ions are seen to contain at least part of the aminopurine group, this is the most likely location of the proton.

Monosodium ADP produces six product ions by CID (Fig. 4(b)). Cleavage **F** occurred by loss of one phosphate group (*m/z* 352.0414, [C_10_H_12_N_5_O_6_PNa]^+^, −1.0 ppm) and cleavage at the **A**/**B** relates to the loss of aminopurine (*m/z* 314.9642, [C_5_H_10_O_10_P_2_Na]^+^, 0.0 ppm), and loss of aminopurine with water (*m/z* 296.9536, [C_5_H_8_O_9_P_2_Na]^+^, 0.1 ppm). **BF** corresponds to an internal product ion formed from the neutral loss of one phosphate and the aminopurine group with the addition of sodium (*m/z* 216.9874, [C_5_H_7_O_6_PNa]^+^, 0.0 ppm) while cleavage at **D** forms a product ion that relates to both phosphate groups (*m/z* 200.9324, [H_4_P_2_O_7_Na]^+^, −0.2 ppm). This shows that the sodium, probably located on the phosphate group, stabilises this group, allowing the aminopurine group to be lost and the diphosphate group to retain a charge. EID of monosodium ADP (Fig. 5(b)) induces bond cleavages mainly concentrated in the ribose region of the ion as with monosodium AMP; the majority being cross‐ring cleavages at the ribose group (**Rib**). The sodium was always retained if the phosphate group was a part of the product ion and was also retained by the lone ribose product ion, **BC**‐H_2_O (*m/z* 119.0103, [C_5_H_4_O_2_Na]^+^, −0.1 ppm) and the lone aminopurine product ion **A** (*m/z* 158.0437, [C_5_H_5_N_5_Na]^+^, 0.1 ppm). These data show that although the sodium is always retained by phosphate‐containing product ions, it can also be retained, although not exclusively, by lone ribose and aminopurine product ions.

CID for disodium ADP (Fig. 4(c)) results in cleavage at nearly all the same bonds as for monosodium ADP, with the product ions retaining both sodiums. Cleavage at **C** was not observed however, as all the product ions from the disodium ADP contain the phosphate. The majority of the EID product ions for disodium ADP (Fig. 5(c)) pertain to cleavages along the phosphate backbone and at the ribose group. In contrast to EID of protonated ADP, all but two product ions contain part of the phosphate group, always with at least one sodium retained. Fewer cross‐ring cleavages at the ribose group were observed (**Rib**), which is analogous to disodium AMP; cleavages are mainly concentrated at the diphosphate group. As this region of the precursor ions is the most likely location of sodium cations, the data suggests that EID is strongly affected by the presence and position of sodium.

CID of trisodium ADP (Fig. 4(d)) resulted in very limited fragmentation, with fewer product ions than monosodium and disodium ADP being formed. The product ions unique to CID of monosodium ADP and disodium ADP are only seen with the inclusion of sodium, showing that the presence of sodium on the diphosphate can weaken the bonds in this region. The loss of H_2_O was observed for the first time (*m/z* 475.9724, [C_10_H_11_N_5_O_9_P_2_Na_3_]^+^, 0.9 ppm) and **F**, as was the loss of one sodium and one phosphate group *(m/z* 374.0241, [C_10_H_11_N_5_O_6_PNa_2_]^+^, 0.3 ppm). EID of trisodium ADP (Fig. 5(d)) yielded fewer product ions than EID of protonated, monosodium and disodium ADP, and induced no cross‐ring cleavage, leading to the proposition that the sodium is associated with the longer phosphate chains, concentrating dissociation in this region of the ion. This is supported by three observations: first that all but one of the sodium‐containing product ions formed are related to the phosphate group; secondly, the formation of **G** (*m/z* 164.9300, [HPO_4_Na_3_]^+^, 0.2 ppm) demonstrates that a single phosphate group can retain three sodiums; and, thirdly, that all the product ions relating to the phosphate groups contain sodium. In general product ions are formed by cleavage at the phosphate groups, always retaining two or three sodiums. The only product ion seen to contain the ribose group was **F** (*m/z* 374.0259, [C_10_H_11_N_5_O_6_PNa_2_]^+^, 5.2 ppm), formed by the loss of one phosphate group and one sodium. Cleavage at **A** forms the lone aminopurine product ion (*m/z* 180.0260, [C_5_H_4_N_5_Na_2_]^+^, 1.9 ppm), showing that the aminopurine group can exist in the disodium form.

### EID and CID of protonated ATP, monosodium ATP, disodium ATP and trisodium ATP

**Figure 6:**
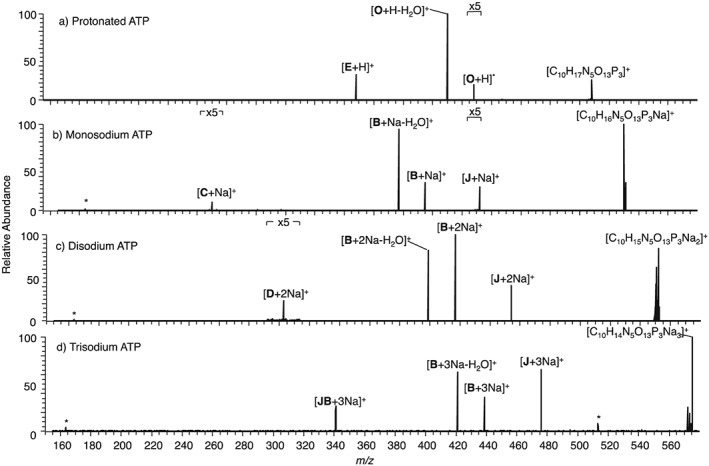
CID of precursor ions with an overall single charge for (a) protonated ATP, (b) monosodium ATP, (c) disodium ATP, and (d) trisodium ATP. A full list of *m/z* values and corresponding molecular formulae is given in the Supporting Information. * indicates instrument noise.

CID and EID of all the ATP precursor ions (Fig. 6) generated similar information to the AMP and ADP precursor ions. EID provided much more information than CID, with the monosodium species again being the most informative. CID of monosodium ATP (Fig. 6(b)) resulted in cleavage at **B**, relating to loss of the aminopurine group, not observed in CID of protonated ATP (Fig. 6(a)), with one sodium, and with one sodium and loss of H_2_O (*m/z* 394.9304, [C_5_H_11_O_13_P_3_Na]^+^, −0.2 ppm and *m/z* 376.9199 [C_5_H_9_O_12_P_3_Na]^+^, −0.1 ppm, respectively) showing that the sodium cation affects the stability in this region of the ion. CID of trisodium ATP (Fig. 6(d)) induced cleavage **JB** (*m/z* 340.9176, [C_5_H_6_O_9_P_2_Na_3_]^+^, 0.4 ppm), which relates to the simultaneous loss of one phosphate group and the aminopurine group. This is the only precursor ion that formed this product ion by CID, probably caused by the location of the three sodium cations affecting the stability of the ribose diphosphate region of the ion.

**Figure 7:**
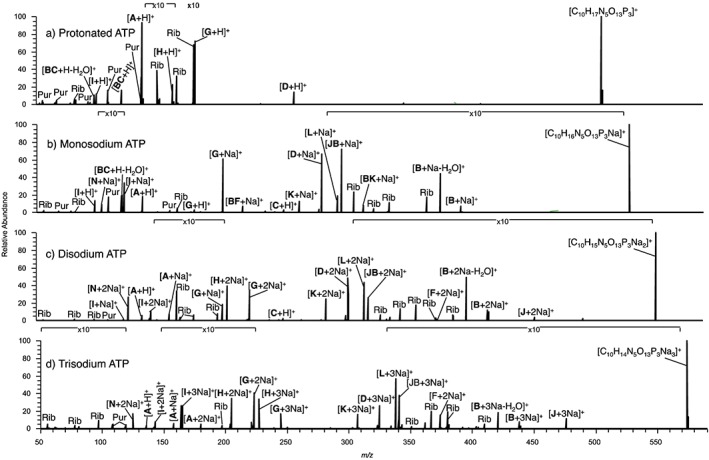
EID of precursor ions with an overall single charge for (a) protonated ATP, (b) monosodium ATP, (c) disodium ATP, and (d) trisodium ATP. A full list of *m/z* values and corresponding molecular formulae is given in the Supporting Information. * indicates instrument noise.

EID of monosodium ATP (Fig. 7(a)) did not result in a product ion relating to the aminopurine ring retaining sodium, as previously seen for monosodium AMP and monosodium ADP. This is probably because the addition of a third phosphate group in the precursor ion biases the location of the sodium cation. EID of trisodium ATP (Fig. 7(d)) resulted in a greater number of small product ions relating to cross‐ring cleavages at the aminopurine group (**Pur**) than for monosodium ATP and disodium ATP (Figs. 7(b) and 7(c)). This is proposed to be because, when three sodiums are included with the precursor ion, there is more chance of one of the sodium cations being located on the aminopurine group, directing some fragmentation towards this group.

## Conclusions

**Figure 8:**
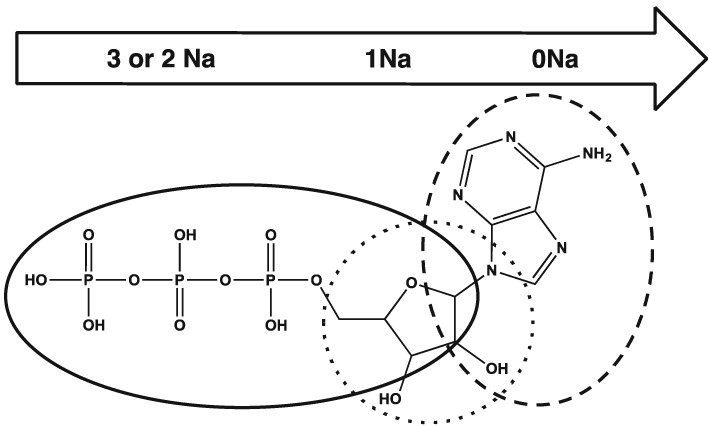
An ATP molecule has been used here to demonstrate the main regions of fragmentation that the ATP ions undergo with EID. For protonated ATP the main regions of fragmentation are shown by the dashed line, for monosodium ATP the dotted line and for di‐ and trisodium ATP the solid line. By extension, this diagram also applies to AMP and ADP.

EID gave significantly more diagnostic information for all the AMP, ADP and ATP precursor ions than CID, where cleavage was mainly limited to the phosphate group. The number of phosphate groups present was not seen to affect the EID product ion spectra, as has been noted for phosphorylated peptides, with common bond cleavages seen for one, two and three phosphate groups.^30^ The number of sodium cations included in the precursor ion had limited effect on the CID product ion spectra. Sodium content did, however, have a dramatic effect on the EID product ion spectra, with the likely locations of the sodium cations directing fragmentation. Regardless of the number of phosphates, when no sodium was included with the precursor ion, EID focused at the aminopurine group suggesting this as the site for protonation. For EID of monosodium precursor ions, dissociation was predominantly seen at the ribose group whereas for EID for disodium and trisodium precursor ions, bond cleavage was generally induced at the phosphate group (demonstrated by Fig. 8 for AMP). The greatest number of product ions for AMP and ADP were observed using EID for the monosodium species, with product ions relating to all regions of the precursor ion. For ATP, however, EID for the trisodium precursor ion formed the most product ions, although the product ion spectra provided limited information as cleavage was mainly limited to the phosphate group. When studying phosphorylated ions, this study indicates that the most diagnostic and prolific tandem mass spectrometric information can be gained by performing EID on a monosodium species. For ions analogous to AMP, ADP and ATP, it is possible to direct EID by fragmenting ions that contain zero to three sodium atoms, enabling targeted study of different groups in the ion.

## Supporting information

Supporting information

Supporting info item
